# A Silicon-Based PDMS-PEG Copolymer Microfluidic Chip for Real-Time Polymerase Chain Reaction Diagnosis

**DOI:** 10.3390/jfb14040208

**Published:** 2023-04-09

**Authors:** Siyu Yang, Qingyue Xian, Yiteng Liu, Ziyi Zhang, Qi Song, Yibo Gao, Weijia Wen

**Affiliations:** 1 Division of Emerging Interdisciplinary Areas, Interdisciplinary Program Office, The Hong Kong University of Science and Technology, Clear Water Bay, Kowloon, Hong Kong; 2 Thrust of Advanced Materials, The Hong Kong University of Science and Technology (Guangzhou), Nansha, Guangzhou 511400, China; 3 HKUST Shenzhen-Hong Kong Collaborative Innovation Research Institute, Futian, Shenzhen 518000, China; 4 Department of Physics, The Hong Kong University of Science and Technology, Clear Water Bay, Kowloon, Hong Kong

**Keywords:** Real-time PCR, PPc-Si chips, PDMS-PEG copolymer, microfluidic chip, molecular diagnosis

## Abstract

Polydimethylsiloxane (PDMS) has been widely used to make lab-on-a-chip devices, such as reactors and sensors, for biological research. Real-time nucleic acid testing is one of the main applications of PDMS microfluidic chips due to their high biocompatibility and transparency. However, the inherent hydrophobicity and excessive gas permeability of PDMS hinder its applications in many fields. This study developed a silicon-based polydimethylsiloxane-polyethylene-glycol (PDMS-PEG) copolymer microfluidic chip, the PDMS-PEG copolymer silicon chip (PPc-Si chip), for biomolecular diagnosis. By adjusting the modifier formula for PDMS, the hydrophilic switch occurred within 15 s after contact with water, resulting in only a 0.8% reduction in transmittance after modification. In addition, we evaluated the transmittance at a wide range of wavelengths from 200 nm to 1000 nm to provide a reference for its optical property study and application in optical-related devices. The improved hydrophilicity was achieved by introducing a large number of hydroxyl groups, which also resulted in excellent bonding strength of PPc-Si chips. The bonding condition was easy to achieve and time-saving. Real-time PCR tests were successfully conducted with higher efficiency and lower non-specific absorption. This chip has a high potential for a wide range of applications in point-of-care tests (POCT) and rapid disease diagnosis.

## 1. Introduction

Polymerase chain reaction (PCR) is a universal analysis methodology in molecular biology. It detects target amplicons by producing copies of specific deoxyribonucleic acid (DNA) molecules or fragments in vitro [1,2]. The fundamental technology was invented by Kary Mullis and has been widely used in prenatal diagnosis [3,4,5], forensic science [6,7], laboratory research in genetics [8,9], and cloning [10] since 1983. Higuchi et al. used the fluorescence of ethidium bromide to monitor DNA amplification in situ with a video camera [11]. This milestone contributed to the subsequent development of quantitative PCR (qPCR, or real-time PCR), in which the fluorescent signal is amplified exponentially as the DNA copying, making both qualitative and quantitative results obtainable [12]. Another significant leap in its related application happened in 1996 when Heid et al. used dual-labeled fluorogenic probes to measure the accumulation of PCR products in a closed tube system, with the advantages of fast analysis, high accuracy and sensitivity, and low labor intensity [13].

In the 21st century, many pandemics, such as Ebola, coronavirus 2019 (COVID-19), and the monkeypox pandemic, have garnered worldwide attention to public health. Real-time PCR and real-time reverse transcription PCR (real-time RT-PCR) have returned to public sight and have become the standard molecular biological methods to diagnose these infectious diseases. The outbreak of COVID-19 has led to a research and industry boom in RT-PCR for detecting the associated virus, severe acute respiratory syndrome coronavirus 2 (SARS-CoV-2) [14,15]. Similarly, monkeypox was declared a public health emergency concern by the World Health Organization (WHO) in 2022 after COVID-19; researchers are continuing to improve the performance of PCR from both the device and reagent sides to deal with the rapidly increased demand and more diverse specimens in clinical diagnosis [16]. Materials for reaction and detection, device-reagent integration, and application convenience are all important factors in improving the efficiency of tests, which also affect their potential for application in point-of-care (POC) diagnostics [17]. We have also reported an all-in-one lyophilized real-time PCR reagent for nucleic acid diagnosis that has the potential to integrate with microfluidic chips, but the fabrication of chips and materials suitable for reagent embedding were yet to be developed at that time [18].

Microfluidics refers to a system that manipulates a very small volume of fluids on the microscale, which was gradually established after the Miniaturized Total Analysis System (μTAS) was first proposed by Manz [19,20,21]. Over the past two decades, microfluidic technology has developed from the initial single-power flow controller to the current multi-power collection. With the features of fast transfer, high surface-to-volume ratio, and low energy consumption, it can achieve fast analysis, automation, and high-throughput screening [21]. Nowadays, microfluidic chips have been used in biomedical fields, including genetic analysis and DNA diagnosis. In 1994, Wilding et al. successfully conducted PCR in a silicon (Si) substrate, which contributed to Kopp et al.’s first continuous-flow PCR chip in 1998 [22,23]. For many existing PCR microchips, bonding glass and patterned Si wafers have been a conventional choice. However, fabrication involves anodic bonding that requires high voltage and temperature (always higher than 400 °C) [24]. In addition, the complex setup and power consumption limit its production capacity. Based on the pattern design of the Si-based system, materials for making microfluidic devices were quickly extended to other biocompatible materials like polydimethylsiloxane (PDMS), glass, and polymethyl methacrylate (PMMA). The high optical transmittance of these cheap materials opens access to an efficient method for chip diagnosis with high resolution, significant sensitivity, and desirable portability [25]. While glass is a suitable material for PCR, its related devices are typically used in microcapillary electrophoresis and have lower testing efficiency and sensitivity than highly integrated microfluidic devices [26,27]. Moreover, compared with PDMS and some soft polymers, glass capillaries are difficult to transform into various shapes and patterns when applied on a chip [28,29].

PDMS is one of the most common and cost-effective substrates with high clarity and a simple manufacturing process among all these materials. Due to its biocompatibility, high transparency, air permeability, adjustable rigidity, and relatively low cost, this material is suitable for a wide range of applications, particularly in micro-patterning [30]. Patterns like microchannels can be replicated on the PDMS surface by soft lithography, with precision down to 2 µm, making it easy to manufacture the desired devices on a small scale and use them as a reactor, sensor, or incubator in the laboratory [31,32,33]. Despite these advantages, PDMS microfluidic devices have problems, such as channel deformation, high evaporation, leaching of uncured oligomers, and absorption of compounds like DNA and drug molecules [31,34,35]. Some research has described methods to bond patterned PDMS with glass to mitigate these problems, using a rigid base as a carrier of the chips [36,37,38]. However, the thermal conductivity of glass (1.13 W/mK) is much lower than Si (163 W/mK) [39]. This restricts the application to research scenes where the heating transfer is not required. Combining PDMS and Si in the microfluidic system can solve the problem better by using both the rapid prototyping of PDMS and the superior thermal conductivity of Si [40]. To date, only a few reports have described the application of PDMS-Si microfluidic chips in the biomedical field. For instance, Wolf et al. conducted a quantitative immunoassay using self-regulating microfluidic networks on PDMS-Si chips [41]. Li et al. performed a fluorescent assay for C-reactive protein (CRP) on their disposable PDMS-Si hybrid chips [42].

However, the permeability and high hydrophobicity of the remaining PDMS surface still hinder the application of these chips. In molecule-related applications, such as biomolecular testing and drug screening, the inherently high hydrophobicity and gas permeability results in the non-specific absorption of molecules and proteins, leading to inaccurate results in these experiments [43]. These predicaments with PDMS applications have prompted researchers to develop alternative materials or modify the original material. There are various PDMS modification techniques that can be used to alter wettability, such as dynamic surfactant treatment, hydrolyzation, and surface and bulk modification with nanomaterials, such as carbon nanotubes and metal nanoparticles [44]. Recent research suggests using hydrogel as an ancillary material for microfluidic devices for cell culture [45,46]. However, it is still challenging to use hydrogel as part of the nucleic acid testing microchip and bond it with hard chip carriers. Furthermore, many of these methods have the disadvantage of reducing transparency and mechanical strength, which can result in surface cracking and increased roughness. Chemical modifiers, such as PMMA, polystyrene (PS), 2-hydroxyethyl methacrylate (HEMA), polyethylene glycol (PEG), and polyvinylpyrrolidone (PVP), are also explored as coating layers through atom transfer radical polymerization (ATRP) to dramatically increase the wettability of PDMS and decrease molecule absorption [47,48,49,50]. Nevertheless, the changed surface property and chemical state of the polymer will affect its bonding strength with the chip carrier, resulting in undesirable leakage and side reactions [51].

A few studies have reported PDMS modification through the pre-mixing method to achieve desirable surface properties. One of the earliest reports by Xiao et al. added Poly (lactic acid)-poly(ethylene glycol) (PLA-PEG) as additives to increase the wettability of PDMS [52]. Later, Zhou et al. modified PDMS by adding polyethylene glycol (PEG) chains with vinyl-group termination to achieve a similar goal [53]. Since PDMS can be copolymerized with PEG to form copolymers, Yao et al., Scofield et al., and Hisyam et al. reported using PDMS-block-PEG as a modifier before curing the material successively and applied the material in microfluidics, CO_2_ separation, and relative permittivity enhancement [54,55,56]. Gökaltun et al. reported a smart polymer that can self-change the wettability over time of contact with water by directly pre-mixing PDMS and PDMS-PEG block copolymer (PDMS-PEG BCP) [57]. This smart copolymer was soon used in drug screening and had huge potential to be used in biological molecule tests and diagnostics [58]. Although the PDMS-PEG copolymer has the significant advantage of lowering the hydrophobicity and molecular absorption of the PDMS surface, few studies have evaluated its optical transmittance properties across a wide range of wavelengths and its bonding strength with silicon to provide a systematic reference for its application in microfluidic diagnostics. Its practical application in this field requires further investigation.

This study explores the application of PDMS-PEG copolymer to real-time PCR. We bonded the patterned material with Si to develop the PDMS-PEG copolymer silicon chip (PPc-Si chip) for real-time PCR diagnosis. PDMS-PEG copolymer was synthesized and made into patterned reaction chips for PCR by soft lithography. The patterned slides were activated by air plasma [59]. Slides were bonded with Si substrates by heating and pressing with an easy-to-reach condition and a time-saving process. We systematically monitored the changes in surface hydrophilicity of the patterned copolymer with varying PEG content over time, evaluated its bonding strength with Si, and tested its optical properties and biological compatibility with real-time PCR. The total cost of the PPc-Si chips for a single test is comparable to that of conventional PDMS chips and much cheaper than silicon-based chips because the mold is reusable, and no extra lithography process is required during fabrication. The device is highly integrated, disposable, and easy to use, making it a high potential for application in point-of-care diagnosis in the future.

## 2. Materials and Methods

### 2.1. Materials

The Polydimethylsiloxane (PDMS) base and curing agent used in this study was Sylgard 184 silicone elastomer kit from Dow Corning. Dimethylsiloxane-(60–70% ethylene oxide) block copolymer was purchased from Gelest. SU-8 2050 photoresist was from Kayaku Advanced Materials Inc. Silicon wafers were provided and cut by the Nanosystem Fabrication Facility of the Hong Kong University of Science and Technology. 

We prepared the basic real-time PCR reaction system with a similar procedure and formula to our previous research [18]. Potassium chloride (KCl), Trizma^®^ base, hydrochloric acid (36.5–38.0%, Tris-HCl), magnesium chloride (MgCl_2_), and Tween-20 surfactant, biological-grade water was purchased from Sigma-Aldrich. Deoxyribonucleotide triphosphates (dNTPs) in the 25 mM solution were ordered from ThermoFisher Scientific. The template we used is the Hepatitis B virus (HBV) plasmids synthesized by GenScript Biotech, which is a standard template for verifying PCR tests and material availability. Primers and TaqMan probes customized for the HBV plasmids were customized from GenScript Biotech following the sequence in Table S1. 6-carboxy fluorescein (6-FAM) was used as a fluorophore, and black hole quencher 1 (BHQ-1) was used as a quencher. Taq polymerase was supplied by Ampliqon A/S. Before the practical test, KCl and MgCl_2_ were dissolved into biological-grade water as 1 M and 0.05 M solutions, respectively. 1 M Tris-HCl buffer solutions at pH 8.09 were prepared with Trizma^®^ base and hydrochloric acid. Primers and probes were mixed into a 100 µM solution (p-mix) for later use. 

### 2.2. Fabrication of PPc-Si Chips

SU-8 photoresist was spin-coated on a 4-inch silicon wafer with a thickness of 250 µm SU-8 manufacturer’s instruction using spin coater (KW-4A, SPI Supplies). Symmetrical patterns of the reaction chip were fabricated on the wafer by photolithography (Desktop Coater AB-M Aligner, A.B. MANUFACTURING, INC.; Hot Plates Cimarec 2 HP46825, Thermolyne) through the sequence of soft-baking, UV-irradiation, post-exposure baking, development, and hard-baking of the photoresist layer. PDMS base and curing agent were mixed on a ratio of 10:1, following adding a demanded volume of PDMS-PEG copolymer. PDMS-PEG copolymer concentrations of the pre-polymer mixture prepared in this study were 0, 0.25 wt%, 0.5 wt%, 0.75 wt%, 1 wt%, and 2 wt%. The mixture was poured on the SU-8-Si mold and degassed in a vacuum for 20 min, followed by the 24-h curing process at 80 °C to obtain patterned PDMS-PEG slides. The patterned slides were cut into 15 × 25 cm blocks. After treated patterns and silicon bases with air-plasma, as mentioned before, the treated surface of the two parts was brought to intimate contact and bonded together at 150 °C, and 0.25 MPa for 3 h. The resulting PPc-Si chips were ready to apply in real-time PCR tests. Another set of conventional PDMS-Si chips was manufactured following the same procedure that only excluded the addition of modifiers.

### 2.3. Optical Transmittance of the Patterned Slides for PPc-Si Chips 

The optical transparency of the modified patterned polymer with various content of PDMS-PEG was determined before the bonding process by ultraviolet-visible spectroscopy (UV-Vis, PerkinElmer Lambda 1050+). We focused the incident light on the area of reaction chambers for each slide and recorded the transmittance in a wide wavelength from 1000 nm to 200 nm. In this study, we treated the transmittance data from 490 to 770 nm as references to choose the proper material for the real-PCR application.

### 2.4. Contact Angle Monitoring of the Patterned Slides for PPc-Si Chips

The area with and without pattern should have the same hydrophilicity. Therefore, the un-patterned part of the modified material was cut into 5 mm × 5 mm blocks for testing to ensure sufficient space for detection. Next, 4 µL water was dropped on the inner surface, and the contact angles were monitored for 10 min by a contact angle meter (Biolin Theta). The optical transmittance and CA results were considered as the basis for choosing the best additive content for the application in this study.

### 2.5. Surface Morphology of the Reaction Channel’s Inner Surface

The inner surface morphology of the PPc-Si chips was examined using a scanning electron microscope (SEM, JEOL-7100). Additionally, the surface roughness of the pattern surface to be bonded with silicon was observed by atomic force microscopy (AFM) imaging (Dimension Icon). An original PDMS slide with the same pattern was tested as a comparison.

### 2.6. The Chemical State of the Reaction Channel’s Inner Surface

The elemental composition and chemical state of the PPc-Si reaction chambers were identified both before and after plasma treatment using X-ray photoelectron spectroscopy (XPS) with an Axis Ultra DLD X-ray photoelectron spectrometer (Kratos). PDMS slides with the same pattern were also tested under the same condition as a reference.

### 2.7. The Bonding Strength of the PPc-Si Chips

The bonding strength for the ready-to-use PPc-Si chips was measured using an air compressor with an in-build barometer (Daertuo XDT550). A needle connected to the compressor was injected through the inlet of the reaction chamber while the chamber outlet was not open. The air pressure increased gradually at a rate of 5 psi/min. The pressure was recorded when the bonding interface cracked, or the material was broken. Both PPc-Si chips and conventional PDMS-Si chips bonded under the same condition were tested.

### 2.8. Real-Time PCR Test in PPc-Si Chips

In this study, we defined the required PCR reaction mix for one reaction in a chamber as 1 reaction unit. For each reaction unit, 0.5 μL 1 M KCl, 0.5 μL 1 M Tris−HCl (pH 8.09), 0.10 μL of 1 M (NH_4_)_2_SO_4_, 0.04 μL of 0.05 M MgCl_2_, 0.16 μL of 25 mM dNTPs, 2 units of Taq polymerase, 0.04 μL of 100 mM p-mix, and 1 × 10^4^ copies of corresponding templates were mixed. Each reaction unit was diluted with PCR-grade water to a final volume of 10 μL and injected into the PPc-Si chips. Chip inlets and outlets were sealed with another plasma-treated PDMS block. Simultaneously, the same reactions were processed in chips made of unmodified PDMS with the same pattern as a reference (Control A). The reactions in chips were performed in the SWM-02 real-time PCR system for PCR chips (Shineway). All reactions were conducted with 45 heating cycles, as we described before [60].

## 3. Results and Discussion

### 3.1. Pattern Design and Fabrication of PPc-Si Chips

**Scheme 1:**
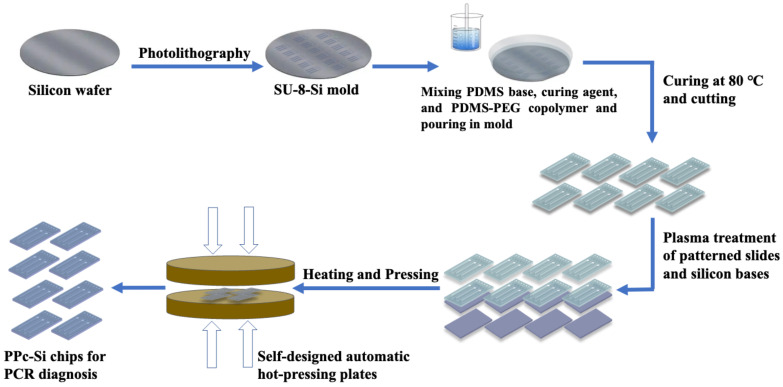
Procedures for fabrication of PPc-Si chips for real-time PCR diagnosis.

We have previously reported on various pattern designs of silicon-based microfluidic chips that were applied in real-time PCR [60,61,62]. This study used the same microchannel design as our previous study on the POC test device. However, we adjusted the size and ratio of the inlets and outlet channels, which was advantageous for conducting soft lithography. Scheme 1 illustrates the fabrication procedure for the PPc-Si chips. The width of the inlets and outlets was reduced to 300 µm, and the depth of all channels was also decreased to 250 µm. The flow channel size was further narrowed down using the high precision of photolithography, ensuring that sample injection could be carried out quickly. After curing for 24 h at 80 °C, we obtained patterns on PDMS-PEG copolymer slides with uniform thickness. According to our experimental result, the insufficient curing time affected the optical transmittance of the cured material (data is not shown). The thickness of the patterned slides was controlled by the weight of the cured mixture, and the surface morphology will be discussed in the following part. The bonding condition was evaluated and confirmed to 150 °C, 0.25 MPa for 1 h. A lower bonding temperature resulted in a longer bonding time, while a higher bonding pressure or a long-time pressing caused the collapse of reaction channels (Figure S1). 

### 3.2. Optical Transmittance of PPSi Chips’ Reaction Channels

The evaluation of optical transparency is crucial to ensure that the material used in the reaction chambers allows for accurate detection of the fluorescent signal produced during the PCR process, which is essential for obtaining reliable results. Previous reports have suggested that PEG content could affect transparency once it exceeds a concentration threshold of around 0.5% [57]. However, few studies have examined the typical wavelength range used in biomolecular diagnosis. The excitation (Ex) and emission (Em) wavelengths of the most used fluorescent dyes in real-time PCR tests are generally from 490 nm to 770 nm, such as 6-carboxyfluorescein (6-FAM, Ex. 495 nm, Em. 519 nm), hexachloro-fluorescein (HEX, Ex. 535 nm, Em. 556 nm), and cyanine-5 (Cy-5, Ex. 650 nm, Em. 670 nm). 

**Figure 1:**
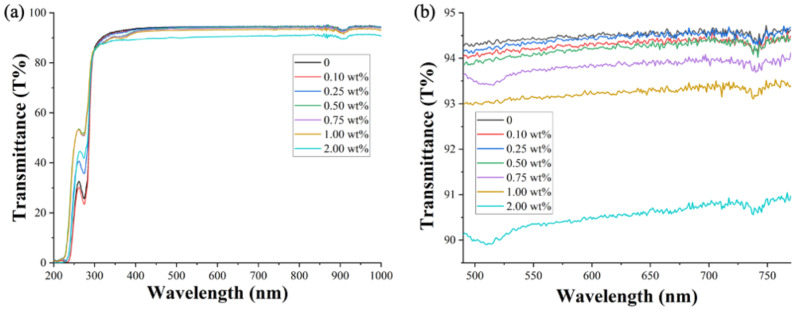
(**a**) Optical transmittance of the synthesized polymer with various content of BCP additives. (**b**) Optical transmittance of the modified polymer on a narrower wavelength range is considered as a reference to find the proper additive content for the real-PCR application.

We first evaluated the optical transmittance of the patterned slides described in this study over a wide range of wavelengths, from 1000 nm to 200 nm (Figure 1a), to provide a comprehensive reference for the optical properties of this copolymer. When the BCP modifier concentration was increased up to 0.75%, the transmittances of each copolymer were comparable above 405 nm. By contrast, the transmittance decreased about 4.25% across the entire tested range after adding 2% of the BCP modifier. Furthermore, the transmittance dropped rapidly for all samples after the wavelength lower than 350 nm, which falls within the ultraviolet range. Fortunately, this range is not typically involved in most common molecular diagnoses and is not relevant to the application discussed in this study. 

After obtaining the wide-range optical transparency reference, we further explored the copolymer’s transmittance within a narrower wavelength range (as mentioned, 770 nm to 490 nm, Figure 1b). With a BCP modifier concentration up to 0.75%, only a 0.8% difference in transmittance was observed. All copolymers exhibited a 0.5% transmittance fluctuation at a wavelength from 730 nm to 750 nm. The downward trend in transparency became more evident when the modifier concentration reached 1.0%. Based on these findings, PDMS-PEG copolymers with a modifier concentration of 0.5% or 0.75% are suitable for fabricating diagnostic devices for biomolecular tests. However, to determine the most appropriate formula, we must consider surface wettability.

### 3.3. Surface Hydrophilicity of the Modified Patterned Slides

**Figure 2:**
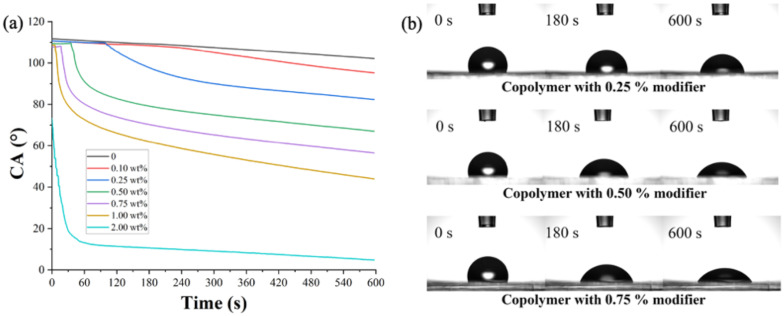
(**a**) Contact angle changes of copolymers with different concentrations of BCP modifier. (**b**) Droplet images were taken after droplets reached the surface of the copolymer pattern with 0.25%, 0.5%, and 0.75% modifiers for 0 s, 180 s, and 600 s during monitoring of the contact angle.

The most suitable material for this application should balance wettability and optical transmittance. To evaluate the wettability of the copolymers, we dropped 4 µL of liquid on the inner area of the chip without patterns and monitored the contact angle (CA) in situ for 10 min. Figure 2a shows the CA changes over time of all tested copolymers. As previous research has described, the hydrophilic chains in the copolymer drive themselves automatically to the surface, creating a hydrophilic surface [57]. According to the graph, this mechanism requires some time for activation, and the activation time becomes shorter with an increase in BCP modifier concentration. The hydrophilic switch started 15 s after contact with water when the copolymer contained 0.75% BCP modifier, while it took around 260 s for copolymers containing 0.1% modifier. The contact angle could reduce to 90° within 45 s and rapidly drop to 80° in 90 s if the concentration of the modifier was higher than 0.75%. These properties reduce the non-specific absorption of molecules and alleviate the hindrance of high air permeability to PDMS application. Figure 2b shows images taken after a droplet was placed on the copolymer containing 0.25%, 0.5%, and 0.75% BCP modifiers for 0 s, 180 s, and 600 s. The complete set of images obtained from all tested copolymer samples is shown in Figure S2. 

After balancing surface hydrophilicity and transparency, we selected a BCP modifier concentration of 0.75 wt% to fabricate the PPc-Si chips and conducted the following characterization and practical tests.

### 3.4. Surface Morphology of the Reaction Channel Inner Surface 

**Figure 3:**
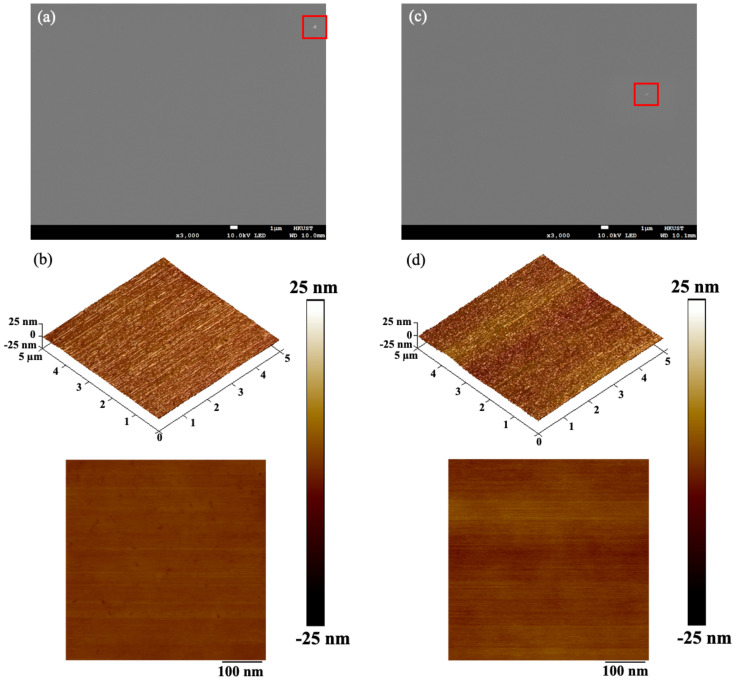
(**a**,**b**) SEM image and 3D & 2D tapping-mode AFM images of the PPc-Si chip’s bonding surface. (**c**,**d**). SEM image and 3D & 2D tapping-mode AFM images of the patterned PDMS slide. Particles marked with red frames are indicators for image focusing.

Surface morphology is crucial for a uniform bonding interface and firm bonding property. Weak surface uniformity can cause unbonded areas and affect bonding strength, increasing the risk of sample leakage during practical tests. We observed the surface morphology and height profile using SEM and SPM-AFM (Figure 3a,b). Compared with the patterned original PDMS slides (Figure 3c,d), PDMS-PEG copolymer had similar surface morphology and roughness. The particles observed in both SEM images, circled by red frames, indicate the fine focus and contrast of the images. No apparent uneven surfaces were found in either SEM image. The height profile of the patterned PDMS-PEG copolymer at a higher resolution showed that the roughness of the copolymer could be controlled within 20 nm, which is comparable to unmodified PDMS. Some nanoscale imperfections were found on the surface of PDMS-PEG, which we deduced may have been caused by some unreacted modifier on the surface. However, the unreacted modifier was removed during the following cleaning process after curing. During the biological test, these defects did not affect the result or efficiency.

### 3.5. Chemical State of the Reaction Channel Inner Surface

**Figure 4:**
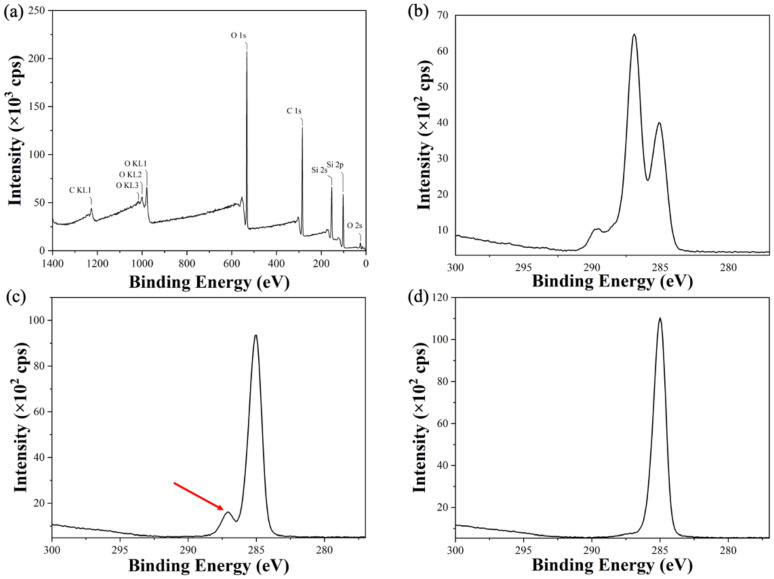
(**a**) XPS spectrum of the PPc-Si chip inner surface (after the plasma is treated during the bonding process). (**b**) The precise spectrum in the bonding energy range of C 1 s of the PPc-Si chip inner surface (after the plasma is treated during the bonding process). (**c**) The precise spectrum in the bonding energy range of C 1 s of the patterned PDMS-PEG copolymer slides (before plasma is treated for bonding). (**d**) The precise spectrum in the bonding energy range of C 1 s of the patterned original PDMS slides (without plasma treatment).

For traditional PDMS blocks, plasma treatment activates silicon and polymer surfaces by increasing the hydroxy group content. Our modification introduced the PEG blockchain in the copolymer, resulting in higher hydroxy group content. The chemical states of patterned slides made with PDMS-PEG copolymer and original PDMS were characterized with XPS (Figure 4). From a macro perspective, the inner surface of the PPc-Si chip had a similar chemical state to plasma-treated PDMS patterned slides (Figure 4a and Figure S3a). However, the spectrum for the bonding energy range of C 1 s with a higher resolution showed a significant difference in C-O bonds and C=O bonds content (the peak at 287 eV and 290 eV, respectively), as shown in Figure 4b and Figure S3b. The amount of C-O and C=O bonds increased significantly in the modified copolymer after plasma treatment. In contrast to PDMS slides without treatment (Figure 4d), the C-O bond peak in Figure 4c, obtained from the copolymer slides without plasma treatment, indicates that the PEG blockchain was well introduced to the copolymer. These optimizations facilitated higher hydrophilicity and bonding strength in the development of diagnostic devices.

### 3.6. Bonding Strength of the PPc-Si Chips 

During conventional real-time PCR tests, 40–45 repetitive heating cycles are processed, including two or three temperature stages from 55 °C to 95 °C for each cycle. This means that the inner pressure of the chip during the test will increase rapidly. We tested the pressure tolerance of the bonded PPc-Si chips and bonded chips with original PDMS patterned slides under the same bonding conditions. Each type of chip was tested four times, and the mean chip failure pressure was calculated. After bonding with the silicon wafer at 150 °C, 0.25 MPa for 3 h, the PPc-Si chips were all well bonded and could sustain a pressure of 4.52 (±0.01) bar, while the conventional PDMS-Si chips could tolerate 2.51 (±0.11) bar. Nevertheless, a 3% bonding failure rate was found for PDMS-Si chips. Furthermore, it should be emphasized that, for most of the PPc-Si chips, the chip failure was not caused by the splitting of the copolymer patterns and silicon wafers. Instead, the patterned copolymer slides themselves were crushed for many tested chips (Figure S4), meaning that the bonding strength should be even higher. This result demonstrates the high potential and advantages of PPc-Si chip applications in biomolecular diagnosis and microfluidic research. The promising bonding strength corresponds to the optimized surface chemical state.

### 3.7. Real-Time PCR Test in PPc-Si Chips

**Figure 5:**
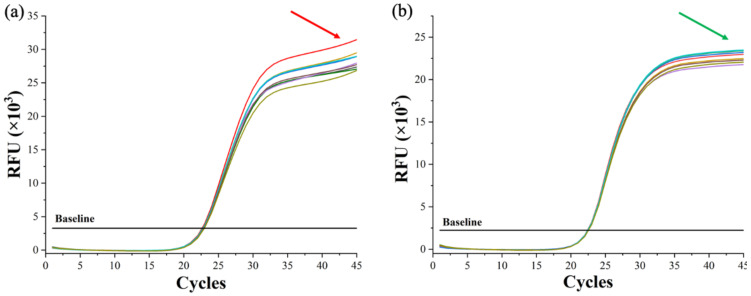
Real-time PCR results for testing of HBV plasmids in (**a**) PDMS-Si chips and (**b**) PPc-Si chips.

The primary goal of this study was to determine the performance of PPc-Si chips in practical real-time PCR tests. Two sets of real-time PCR were conducted simultaneously on PPc-Si chips and conventional PDMS-Si chips to detect HBV plasmids. The same experiment was repeated several times, and nine curves from each set were selected and shown in Figure 5. Both chips provided clear amplification curves. PDMS-Si chips had an average quantification cycle (Cq) value of 22.80 (±0.16), while PPc-Si chips achieved a mean of 22.41 (±0.08). The Cq values of each test in both chips are compared in Table S2. After the exponential amplification stage, there should be a plateau stage in the last 3 to 5 cycles [63,64]. As pointed out by the red arrow in Figure 5a, the relative fluorescent units kept rising after the plateau stage, and the growth rate increased in the last two cycles. This phenomenon is often attributed to the evaporation of reacting liquid or undesired absorption during the reaction process [65,66]. Both reasons may lead to serious consequences, such as inaccurate diagnosis or misdiagnosis and biological contamination leakage. 

The use of the PPc-Si chip eliminated the abnormal plateau stage, which corresponds to hydrophilicity, optimized gas permeability, and reduced molecule absorption. The average 0.5 cycle early in the Cq values also revealed the reduced biomolecular absorption of the PPc-Si chip compared to traditional PDMS-Si chips (Table S2). The lower relative fluorescence units (RFU) were attributed to the decrease in optical transmittance of the modified copolymer, despite the low clarity reduction rate caused by the modifier, as mentioned earlier. Nevertheless, the fluorescent signal was still sufficient to obtain a high-accuracy testing result. More methods should be explored to increase the transparency of the PDMS-PEG copolymer, such as secondary additives or adjustments to curing conditions. 

## 4. Conclusions

A silicon-based PDMS-PEG copolymer microfluidic chip, the PPc-Si chip, was developed for biomolecular diagnosis. PDMS-PEG copolymer was synthesized with varying content of the BCP modifier. Optical transmittance and contact angle of copolymers with various modifier content were observed. The transmittance decreased by about 4.25% at the entire tested wavelength range after adding 2% of the BCP modifier. In contrast, only about 0.8% of transmittance reduction occurred with a BCP modifier concentration of up to 0.75%. The wide-range evaluation of transmittance provided a reference for its optical property study and application in optical-related devices. The contact angle reduced to 90° within 45 s and rapidly dropped to 80° in 90 s when the modifier concentration was higher than 0.75%. The hydrophilicity switch started 15 s after contact with water at this concentration. The surface morphology of the patterned copolymer was similar to traditional PDMS patterns, with the roughness controlled within 20 nm. XPS results illustrated the high bonding strength with silicon wafers up to 4.52 bar. Real-time PCR tests for HBV were successfully conducted on PPc-Si chips with improved efficiency and optimized curve shapes, which benefited from the optimized surface properties and diminished non-specific absorption of molecules.

Overall, the novel microfluidic chip developed in this study opens access to integrate silicon substrates with modified PDMS copolymers, extending the application of PDMS microfluidic devices that were previously hindered by high hydrophobicity and molecular absorption. The fabrication procedure is simple, cost-effective, and time-saving, and the bonding conditions for the chips are mild. This integrated chip shows high potential for a wide range of practical applications and significantly benefits point-of-care testing for food safety, environmental research, and disease diagnosis.

## Data Availability

The data that supports this study are available from the corresponding author upon reasonable request.

## Supplementary Materials

The following supporting information can be downloaded at: https://www.mdpi.com/article/10.3390/jfb14040208/s1, Table S1. Sequences of primers and probes used in this study to test the PPc-Si chips; Figure S1. Collapsed microfluidic chips after being pressed at 0.5 MPa. The inner surface of the reaction channel was incorrectly bonded with the silicon base; Figure S2. Droplet images shot after the droplets reach the surface of the copolymer with different concentrations of BCP modifier for 0 s, 180 s, and 600 s (from left to right, respectively) during the monitoring of the contact angle; Figure S3. a. XPS spectrum of the original patterned PDMS after treated with plasma. b. The precise spectrum in the bonding energy range of C 1 s of the patterned original PDMS slides after plasma treatment; Figure S4. The chip failure was caused by crushing the patterned copolymer slides. Table S2. Comparison of the real-time PCR results from PPc-Si chips and conventional PDMS chips.
