# Supplementary material for: A Silicon-Based PDMS-PEG Copolymer Microfluidic Chip for Real-Time Polymerase Chain Reaction Diagnosis
Source: J Funct Biomater. 2023 Apr 9;14(4):208. doi: 10.3390/jfb14040208 (PMC10143339; doi:10.3390/jfb14040208)
Supplement: Supplementary file 1 [file jfb-14-00208-s001.zip › jfb-2316234-supplementary.pdf]

A Silicon Based PDMS-PEG Copolymer Microfluidic Chip for Real-time Polymerase Chain Reaction Diagnosis

Supplementary Materials

Table S1. Sequences of primers and probes used in this study to test the PPc-Si chips.

Primers and probes	Sequences (5′ to 3′)
C-HBV2 Pb478	6-FAM-ACTCCCTCGCCTCGCAGACGA-BHQ-1
C-HBV2 F382	ACTCCTCCAGCTTATAGACCACCA
C-HBV2 R518	CGACGCGGCGATTGAGA

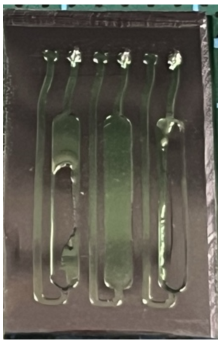

Figure S1. Collapsed microfluidic chips after being pressed at 0.5 MPa. The inner surface of the reaction channel was incorrectly bonded with the silicon base.

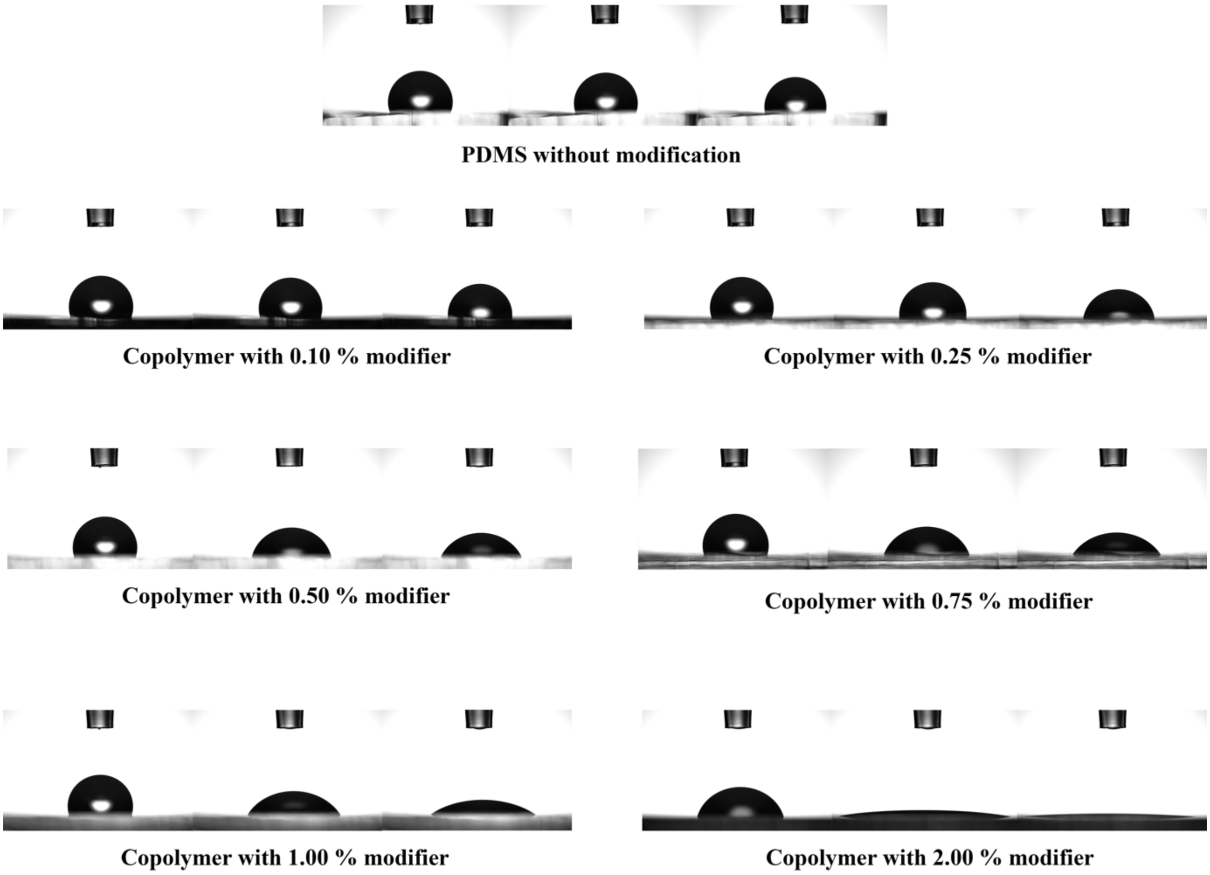

Figure S2. Droplet images taken after droplets reached the surface of copolymer with different concentrations of BCP modifier for 0 s, 180 s, and 600 s (from left to right, respectively) during monitoring of the contact angle.

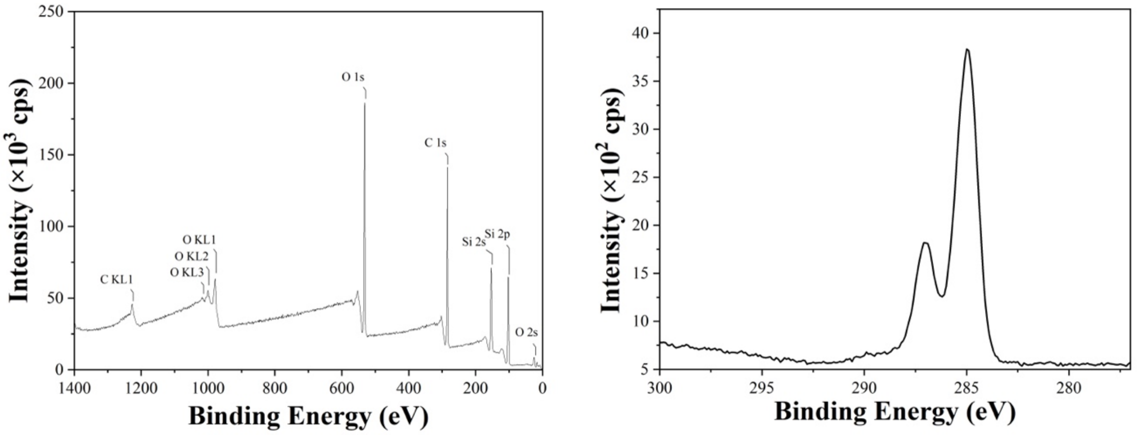

Figure S3. a. XPS spectrum of the patterned original PDMS after treated with plasma. **b.** The precise spectrum in the bonding energy range of C 1s of the patterned original PDMS slides after plasma treatment.

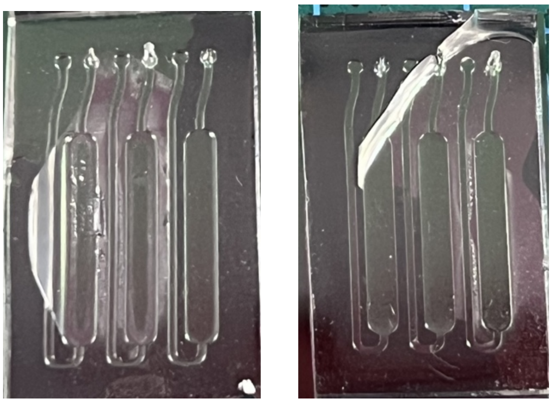

Figure S4. The chip failure caused by crushing the patterned slides.

Table S2. Comparison of the real-time PCR results from PPc-Si chips and conventional PDMS chips.

No.	PPc-Si chips	Conventional PDMS chips
**1**	22.38	22.84
**2**	22.39	22.89
**3**	22.43	22.79
**4**	22.43	22.62
**5**	22.49	22.75
**6**	22.49	22.76
**7**	22.43	22.72
**8**	22.52	22.87
**9**	22.53	22.96
